# Global Transcriptome Analysis During Adipogenic Differentiation and Involvement of Transthyretin Gene in Adipogenesis in Cattle

**DOI:** 10.3389/fgene.2018.00463

**Published:** 2018-10-18

**Authors:** Hanfang Cai, Mingxun Li, Xiaomei Sun, Martin Plath, Congjun Li, Xianyong Lan, Chuzhao Lei, Yongzhen Huang, Yueyu Bai, Xinglei Qi, Fengpeng Lin, Hong Chen

**Affiliations:** ^1^College of Animal Science and Technology, Northwest A&F University, Yangling, China; ^2^College of Animal Science and Technology, Yangzhou University, Yangzhou, China; ^3^Animal Genomics and Improvement Laboratory, United States Department of Agriculture-Agricultural Research Service, Beltsville, MD, United States; ^4^Animal Health Supervision in Henan Province, Zhengzhou, China; ^5^Biyang Bureau of Animal Husbandry of Biyang County, Biyang, China

**Keywords:** adipocyte differentiation, alternative splicing, cattle, differentially expressed genes, transthyretin, transcriptome analysis

## Abstract

Adipose tissue plays central role in determining the gustatory quality of beef, but traditional Chinese beef cattle have low levels of fat content. We applied RNA-seq to study the molecular mechanisms underlying adipocyte differentiation in Qinchuan cattle. A total of 18,283 genes were found to be expressed in preadipocytes and mature adipocytes, respectively. 470 of which were significantly differentially expressed genes (DEGs) [false discovery rate (FDR) values < 0.05 and fold change ≥ 2]. In addition, 4534 alternative splicing (AS) events and 5153 AS events were detected in preadipocytes and adipocytes, respectively. We constructed a protein interaction network, which suggested that collagen plays an important role during bovine adipogenic differentiation. We characterized the function of the most down-regulated DEG (*P* < 0.001) among genes we have detected by qPCR, namely, the transthyretin (TTR) gene. Overexpression of TTR appears to promote the expression of the peroxisome proliferator activated receptor γ (PPARγ) (*P* < 0.05) and fatty acid binding Protein 4 (FABP4) (*P* < 0.05). Hence, TTR appears to be involved in the regulation of bovine adipogenic differentiation. Our study represents the comprehensive approach to explore bovine adipocyte differentiation using transcriptomic data and reports an involvement of TTR during bovine adipogenic differentiation. Our results provide novel insights into the molecular mechanisms underlying bovine adipogenic differentiation.

## Introduction

In livestock (cattle, sheep, pigs, and others), there are four major adipose depots – visceral, subcutaneous, intermuscular, and intramuscular fat tissues – which develop by a process called adipogenesis (Fernyhough et al., 2005; Hausman et al., 2009). Their occurrence during ontogeny follows the sequence of visceral tissue first, followed by subcutaneous, intermuscular, and eventually intramuscular fat tissues (Hausman et al., 2009). In cattle, adipocytes are formed in visceraland subcutaneous tissues at the start of the second trimester during gestation (Fève, 2005; Gnanalingham et al., 2005; Muhlhausler et al., 2007). After 180 days of gestation, adipocytes are barely detected in the intermuscular fat (Du and Dodson, 2011). From an agricultural perspective, the amount and distribution of fat in beef cattle and other farm animals determines the quality of the overall carcass and meat quality (Powell and Huffman, 1973; Wheeler et al., 1994; Lozeman et al., 2001). Research on bovine fat tissue formation, therefore, not only provides general insights into the regulatory processes underlying mammalian adipogenesis, but, also provides invaluable information for breeding programs aimed at improving the beef.

Until now, studies using preadipocyte cell lines obtained from humans (Green and Kehinde, 1975) and mice (Green and Kehinde, 1976; Negrel et al., 1978) have identified several factors that play a role during adipogenesis, such as PPARγ, the CCAAT/enhancer-binding protein (CEBP) family, growth factors, and other cell factors (McLaughlin et al., 2007). In contrast to the wealth of knowledge obtained from studies on murine and human cell lines, the regulatory mechanisms of bovine have received comparatively little attention. Our study on bovine adipogenesis was motivated by the observation that Chinese beef cattle have a low intramuscular fat content and insights into the molecular mechanisms involved in the regulation of adipogenesis may help during assisted breeding programs that use characterization of potential breeding stock to improve overall meat quality.

In recent years, high-throughput sequencing of coding and non-coding RNAs (RNA-seq) has been increasingly applied to unravel the complex molecular mechanisms underlying various biological processes (Mortazavi et al., 2008). Using RNA-seq allows linking changes in gene expression (i.e., mRNA abundance) to the physiological state of tissue under examination, provides comprehensive data from which global gene networks can be constructed, and identifies novel transcriptional unites altered during developmental processes or diseases (Grant et al., 2011). Several studies have reported on transcriptional characteristics related to adipose tissue development using oligo (dT) primers to sequence mRNA. As a consequence, transcripts without a polyA-tail and partial degraded mRNAs are underrepresented in previous studies on mammalian asipogenesis (Zhou et al., 2014; Huang et al., 2017; Zhang Y.Y. et al., 2017). The Ribo-Zero RNA-seq method provide an alternative that can detect mRNAs with and without polyA-tail from intact or fragmented RNA samples, which overcomes the shortcomings of traditional RNA-seq.

In this study, we selected TTR gene, which is significantly differently expressed between preadipocytes and adipocytes, as candidate to primarily explore its role in bovine adipogenic differentiation. TTR is one member of prealbumins (Ingbar, 1958). It is highly conserved among vertebrate species (Schreiber and Richardson, 1997; Power et al., 2000). It is a famous carrier protein, which helps to transport thyroid hormones in plasma and cerebrospinal fluid. It also transport a binding part for RNA binding protein 4 (Raz and Goodman, 1969; Vieira and Saraiva, 2014). Except that, TTR is involved in some intracellular processes, such as proteolysis (Costa et al., 2008), nerve regeneration (Fleming et al., 2007, 2009), and autophagy (Vieira and Saraiva, 2013). Most researches about TTR is focused on the association between its mutations and diseases, such as amyloidosis (Jacobson et al., 1997; Coelho et al., 2013), hereditary (Coelho et al., 2013), and hyperthyroxinemia (Saraiva, 2001). Several reports display serum TTR is associated with body mass and obesity (Cano et al., 2004; Klöting et al., 2007; Zemany et al., 2014). However, little is known about the molecular terms the role of TTR in adipose development.

The aim of our present study were to compare the transcriptome profiles of preadipocytes and adipocytes using Ribo-Zero RNA-seq to gain insight into the potential molecular mechanisms underlying preadipocyte differentiation in beef cattle. For our study we used Qinchuan cattle, which are famous beef cattle native to China. The results of our study not only serve as a basis for further studies on bovine adipogenesis in China.

## Materials and Methods

All experiments were approved by the Review Committee for the Use of Animal Subjects of Northwest A&F University. All experiments were performed in accordance with relevant guidelines and regulations.

### Bovine Preadipocyte Isolation, Adipogenic Differentiation, and Treatment

In brief, tissue separation method was carried out to isolate the preadiocytes from fat tissue. The inguinal subcutaneous fat was separated from two 1 year old male Qinchuan cattle immediately after they were slaughtered. These cattle were raised and slaughtered in Qinbao Animal Husbandry Co., Ltd, which is a cattle breeding and slaughtering corporation in Xi’an, Shaanxi province. The adipose tissue was transported to the laboratory in phosphate-buffered saline (PBS) with 300 IU/mL penicillin and 300 μg/mL streptomycin at room temperature. The tissue was successively washed with 70% alcohol for 1–2 min and three times with PBS. The outer layer was separated and remainder was twice washed with PBS including 100 IU/mL penicillin and 100 μg/mL streptomycin, and finely chopped into 1-mm^3^. The tissue pieces were evenly placed onto the bottom surface of a culture bottle containing growth medium (GM), which contains high glucose DMEM with 20% fetal bovine serum (FBS), 100 IU/mL penicillin and 100 μg/mL streptomycin. The samples were then incubated at 37°C in a humidified atmosphere containing 5% CO_2_. The GM should be replaced every other day.

After reaching 100% confluence, preadipocyte differentiation was induced by adipogenic agents composing of 10 μg/mL insulin, 0.5 mM 3-isobutyl-1-methylxanthine (IBMX) and 1 μM dexamethasone for 2 days. The cells were then incubated with 10 μg/mL insulin, changing the medium every second day.

For Oil Red O staining, cells were washed with PBS and then fixed with 4% paraformaldehyde for 1 h at 4°C. After washing twice with PBS, the cells were stained with Oil Red O solution (0.3% Oil Red O, 60% isopropanol, and 40% PBS) for 20 min. In order to evaluate the amount of lipid droplets, isopropyl alcohol was used to elute the lipid droplets, and then the OD values were measured by UV spectrophotometer at 490 nm.

In TNFα treatments, mature adipocytes were treated with different concentrations of TNFα (2.5, 5, 10, and 20 ng/mL) for 12 h after being cultured with serum-free medium. Then cells were collected for RNA extraction and cDNA preparation.

### RNA Extraction and cDNA Libraries Construction

Samples, including two groups of cells (two wells of undifferentiated adipocytes and two wells of adipocytes cultured by adipogenic agents for 13 days), were collected for sequencing. Consequently, four cDNA libraries were constructed (preadipocyte-1, preadipocyte-2, adipocyte-1, and adipocyte-2). Total RNA was extracted from cells using TRizol reagent (Life Technologies, United States) according to the instructions. Quality was monitored by NanoDrop ND-1000 and Agilent Bioanalyzer 2100 (Agilent Technology, United States). The RNA was purified by RNeasy Micro kit (QIAGEN, Germany) and RNase-Free DNase Set (QIAGEN, Germany). rRNA was removed using Ribo-Zero rRNA Removal Kits (Epicentre, United States) and then rRNA-depleted mRNA was fragmented as a template for the first- and second-strand cDNA synthesis. These short fragments were purified with Quit dsDNA HS Assay Kit (Invitrogen, United States) and connected with different ligate adapters for sequencing.

### High-Throughput Sequencing and Data Analysis

Each of the four libraries was sequenced by Shanghai Biotechnology Corporation (Shanghai, China) using Illumina HiSeq^TM^ 2500. The sequencing quality was checked using FastQC (Andrews, 2015). Pre-processing and assembly of raw sequence data, including removal of the adapter sequences, low-quality sequences, sequences shorter than 20 nucleotides, and other nucleotides, were performed using the FASTX-Toolkit (version 0.0.13). Clean reads were then mapped to the Bos Taurus genome^1^ using Tophat with three base mismatches allowed. The Cufflinks program (version 2.1.1) was used to calculate the expression of transcripts. Data was normalized by calculating the FPKM for each gene. The mapping results were compared to the known gene recorded in the database using Cufflinks compare (Henze et al., 2008). Those not overlapped with known genes were regarded as potential novel genes.

### Identification of AS

The astalavista (version: 3.2), a server extracts and displays AS events from a given genomic annotation of exon-intron gene coordinates, was applied to detect the AS sites and models in final transcriptome assembly, which was achieved using cufflinks (Foissac and Sammeth, 2007; Sammeth et al., 2008). And then mixture-of-isoforms (MISO) was carried out to quantitate the expression level of alternatively spliced isoforms.

### GO Annotation and Pathway Enrichment Analysis of DEGs and Construction of Protein Interaction Network

The fold-change and *P*-value, which was decided by FDR using Fisher-test, of each gene in two groups were calculated. The fold-change ≥ 2 and FDR < 0.05 were considered as the threshold to distinguish the significance of gene expression differences. GO^2^ and KEGG^3^, which are evaluated by the DAVID software (Huang et al., 2009a,b), are major bioinformatics methods to unify the representation of genes and gene products attribute across all species (Ashburner et al., 2000). The corrected *P*-value ≤ 0.05 was taken as the significance threshold. The DEGs that were enriched to the top three pathways were clustered in STRING 10.0 (Szklarczyk et al., 2015) and used to construct a protein interaction network by Cytoscape 3.4 (Shannon et al., 2003).

### qPCR

To validate the high-throughput sequencing data, in addition to the cDNA libraries used in the RNA-seq, two more libraries in each group were constructed, qPCR was performed to confirm the transcriptional levels of DEGs that had been identified as being significantly differently expressed between undifferentiated and differentiated adipocytes. Total RNA was extracted from undifferentiated and differentiated adipocytes using Trizol kit (Takara, Japan). cDNA was synthesized as template in qPCR according to PrimeScript RT Reagent Kit (Perfect Real Time) (Takara, Japan). Glyceraldehyde-3-phosphate dehydrogenase (GAPDH) gene was chosen as internal control. The primers used are shown in **Supplementary Table S5**. PCR was carried out in a CFX96TM Real Time detection system with SYBR premix ExTaq II (TaKaRa, Japan) following manufacturer’s instruction. All samples were measured in triplicate and a negative control with water as template was included. The relative expression ratios were calculated with the following formula 2^−ΔΔCt^ as Schmittgen and Livak described (Schmittgen and Livak, 2008).

### Construction and Cell Transfection

The CDS region of bovine *TTR* gene (GenBank Accession Number: NM_173967.3) was cloned from adipocytes using the forward primer: 5′-CGGGGTACCATGGCTTCCTTCCGTCTGTTCC-3′ and the reverse primer: 5′-GCTCTAGATCACGCCTTGGGACTGCTGA-3′, then recombined into the pcDNA3.1 (+) plasmid vector between the *Kpn*I and *Xba*I (TaKaRa, Dalian, China) restriction sites to construct the overexpression vector of bovine *TTR* gene (OV-TTR). The empty pcDNA3.1 (+) plasmid without any insertion fragment was set as negative control (OV-NC). The plasmids were transfected into cells using Lipofectamine 2000 (Invitrogen, United States) according to the manual. The cells were seeded into 12-well plates in triplicate and transfected with OV-TTR, OV-NC on 7 day after adipogenic induction, respectively. On 9 day and 11 day post-adipogenic induction, the cells were collected for RNA and protein extraction using RNAplus (Takara, Japan) and radio immunoprecipitation assay (RIPA) with 1 mM phenylmethanesulfonyl fluoride (PMSF) (Solarbio, China), respectively.

### Western Blot

Proteins were separated in 12% SDS-PAGE gel. The primary antibody, mouse monoclonal anti-PPARγ2 was purchased from Santa Cruz Biotechnology (Santa Cruz, CA, United States), mouse monoclonal anti-FABP4 and mouse monoclonal anti-β-actin were obtained from Sangon (Shanghai, China). The second antibody was horseradish peroxidase-conjugated ECL goat anti-mouse IgG. After being treated with ECL Plus (Solarbio, China), the protein bands were figured by ChemiDoc XRS + system (Bio-Rad Laboratories, United States).

### Statistical Analysis

The significance of differences in expression level were calculated by Student’s *t-*test in SPSS software (Version 20). The results were presented as mean ± SE (Standard Error), and *P*-value < 0.05 was considered statistically significant.

## Results

### Bovine Adipocytes Culture

After the tissue pieces were seeded for 2 days, bovine preadipocytes gradually spread out around the tissue pieces and showed spindle or polygon morphologies. Cells proliferated rapidly at days 4 and 5. On the 8th day, the confluence reached at 100% (**Supplementary Figure S1A**). The primary adipocytes were then collected for subculture.

After the preadipocytes had proliferated to 100% confluence, the cells were induced by adipogenesis agents. On the 3rd day of differentiation, lipid droplets began to form and increased gradually in numbers. Starting around 6th day, the size of lipid droplets became bigger and bigger. Oil Red O staining confirmed the formation of lipid droplets on the 13th day after inducing differentiation (**Supplementary Figures S1B,C**).

In order to validate the cultured cells were adipose cells, the expression of preadipocyte marker gene, preadipocyte factor 1 (Pref-1) (Wang et al., 2006) was validated by using reverse transcriptional PCR (RT-PCR) (Primers in **Supplementary Table S5** and **Supplementary Figure S1D**). In addition, mRNA expression profiles of four marker genes of mature adipocytes, PPARγ, CEBPα, FABP4, and lipoprotein lipase (LPL) (Ntambi and Young-Cheul, 2000) were evaluated by quantitative real-time quantitative PCR (qPCR) (**Supplementary Figure S1F**). As predicted, Pref-1 was mainly expressed in preadipocytes, while the other four genes were more expressed after differentiation.

### Deep Sequencing of RNAs

Before Ribo-Zero ribonucleic acid sequencing, the RNA samples extracted from two preadipocyte samples and two adipocyte samples were examined firstly. The OD260/OD280 ratios of these four samples were all >1.8 and the amount of them were enough for sequencing. Accordingly, four cDNA libraries were successfully constructed; from each of these libraries, more than 74 M clean reads were obtained. The ratios, bases of quality ≥20 to all bases of sequencing, were all >96%, suggesting that our sequencing results were reliable and suitable for in-depth statistical analysis.

Averages of up to 95.75% (preadipocytes) and 97.6% (mature adipocytes) clean reads ratio were acquired. Correspondingly, average 90 and 89.4% mapping ratio were achieved, respectively. Among them, 83.1 and 82.7% of reads were uniquely mapped to reference genome (CNCI Bos_taurus_4.6.1), respectively. More than 89% of the clean reads were mapped to genic regions of the genome (**Supplementary Figure S2**).

### Gene Expression Patterns

**FIGURE 1:**
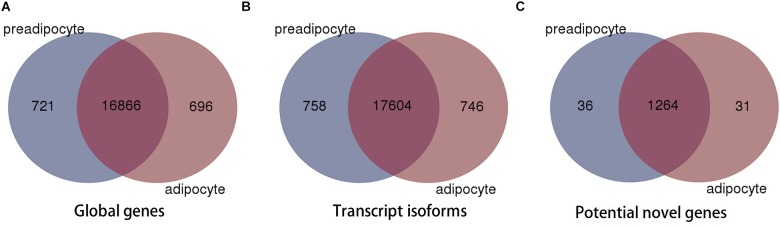
Venn diagram of global genes **(A)**, transcript isoforms **(B)**, and potential novel genes **(C)**.

We determined global levels of gene expression profiles in preadipocytes and adipocytes and used FPKM-value (fragments per kilobase of exon model per million mapped reads) to compare expression profiles between different genes and between both cell types. Altogether, we found 18,283 genes to be expressed in preadipocyte and adipocytes. We also detected genes that were specifically expressed at only one developmental stage, 779 of which were unique to preadipocytes and 1,082 to adipocytes (**Figure 1A**). After mapping, 1,331 genes could not be mapped to known genes and were assembled as potential novel genes (**Figure 1C** and **Supplementary Table S1**). 71.5% of them had only one exon, and 68.1% of them were shorter than 2 Kb (**Supplementary Figure S3**).

Alternative pre-mRNA processing can produce multiple transcript with distinct or similar functions from a single genomic locus. These gene isoforms have important regulatory functions in the development of diverse cell types (Black, 2003; Kim et al., 2004). We evaluated the occurrence and abundance of transcript isoforms, and detected a total of 19,108 transcript isoforms (18,362 in preadipocytes and 18,350 in adipocytes, respectively) were detected. 758 transcript isoforms were only presented in preadipocytes and 746 were unique to adipocytes, respectively (**Figure 1B**).

### AS Detection

Among all the genes detected, up to 6411 genes, approximately 32% were found to be alternatively spliced. However, single sample analysis revealed that 938 genes were uniquely alternative spliced in adipocyte, while the AS events of 881 genes only happened in preadipocytes. It was noteworthy that the number of genes undergoing AS events on chromosome 3 (1105) was the largest.

**FIGURE 2:**
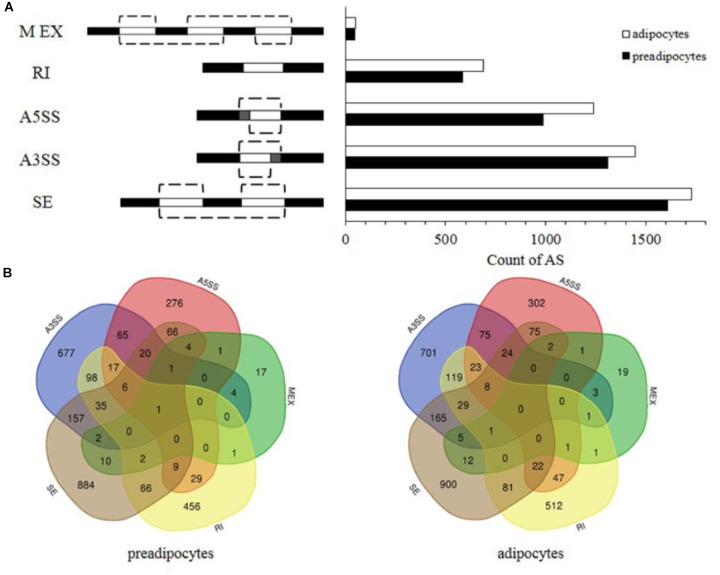
Comparison of AS patterns in preadipocytes and adipocytes. **(A)** Number of AS events in preadiopocytes and adipocytes. **(B)** Overlap of five types of AS genes in preadipocytes and adipocytes.

Previous study revealed that skipped exon (SE), alternative 3′ SS selection (A3SS), alternative 5′ SS selection (A5SS), retained intron (RI), and mutually exclusive exons (MEX) are the majority AS events (Li et al., 2013). The corresponding values were 1607, 1309, 987, 584, and 47 in preadipocytes, respectively, and 1728, 1447, 1240, 687, and 51 in adipocytes, respectively (**Figure 2A**). SE was the most frequent in both preadipocytes and adipocytes and AS events occurred more frequently in adipocytes than they did in preadipocytes. Interestingly, Venn diagrams illustrate a series of overlapping genes among five types of AS in both preadipocytes and adipocytes, and their distribution is different between preadipocytes and adipocytes. Furthermore, 11 and no genes exhibited all types of AS in the preadipocytes and adipocytes, respectively (**Figure 2B**).

### Function Annotation of DEGs and Construction of Protein Interaction Networks

**FIGURE 3:**
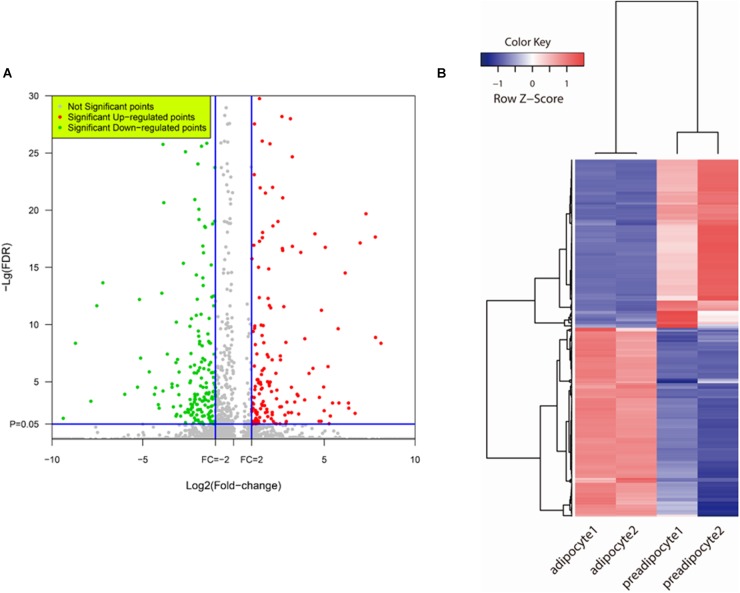
DEGs detection. **(A)** Volcano plot of bovine adipogenesis related DEGs expression profiling. Red plots represent genes up-regulated significantly and green plots represent genes down-regulated significantly. **(B)** Heat map of DEGs. Hierarchical cluster and cluster tree analysis was used to create the dendrogram. The heat map compares fold change patterns of the most highly DEGs in preadipocytes and adipocytes. Each line represents one gene. Left two panels: adipocyte; Right two panels: preadipocytes. Red indicates high expression; blue indicates low expression.

Based on FDR values < 0.05 and fold change ≥ 2, of the 470 DEGs shown in Figure 244 down-regulated and 226 up-regulated genes showed significant changes in transcript abundance when comparing expression profiles of adipocytes to those of preadipocytes (**Figure 3A** and **Supplementary Table S2**). The strength of change in gene expression (fold changes) of 100s of DEGs were ≥ 10, alluding to their potential involvement in bovine preadipocyte differentiation (**Figure 3B**). Also, the expression levels of 817 AS isoforms were different among adipocytes and preadipocytes.

**FIGURE 4:**
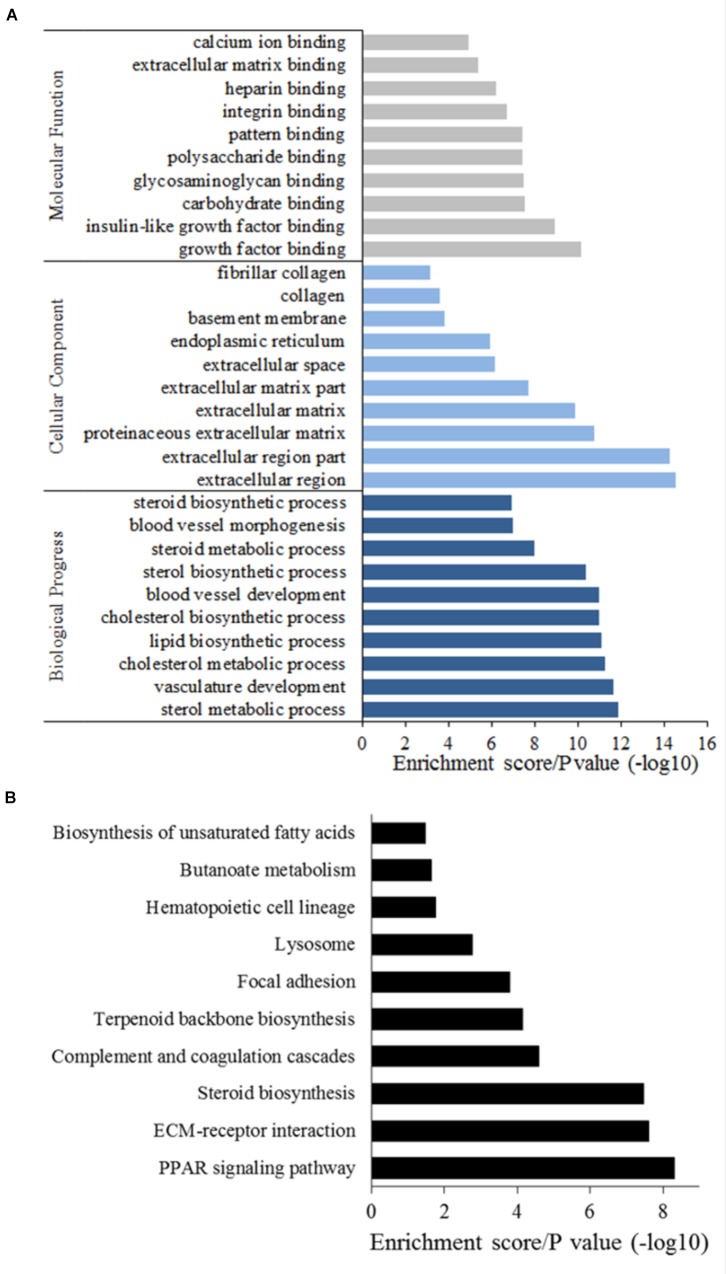
GO annotation **(A)** and KEGG analysis **(B)** of DEGs.

To gain into the potential biological processes related to bovine adipogenesis in which DEGs are involved, we performed analysis of Gene Ontology (GO) and Kyoto Encyclopedia of Genes and Genomes (KEGG) pathways. In total, 305 GO terms were assigned to the 470 DEGs, and 219 terms were significantly enriched (*P* < 0.05). Among these 219 terms, 150 were corresponding to biological process, 24 to cellular component, and 45 to molecular function, respectively (**Supplementary Table S3**). Considering biological processes involved DEGs, we found sterol metabolic processes, vasculature development, and cholesterol metabolic processes, and DEGs were mainly enriched in extracellular components (**Figure 4A**). Interestingly, the top 10 categories related to biological processes in which DEGs were enriched were all related to adipose tissue development, obesity, and energy metabolism (**Supplementary Table S3**). Among these, the category, lipid biosynthetic processes was remarkable as the greatest number (*n* = 26) of DEGs were enriched in this category, including LPL, fatty acid synthase (FASN), 24-dehydrocholesterol reductase (DHCR24) and fatty acid desaturase 2 (FADS2). Considering carbohydrate binding, which is associated with fat metabolism, we detected an enrichment of C-type lectin domain family 3, member B (CLEC3B), fibronectin 1 (FN1), thrombospondin 1 (THBS1), pleiotrophin (PTN), and others. Most of these DEGs were up-regulated during the differentiation of preadipocytes into mature adipocytes.

Considering KEGG pathways, we found a total of 196 pathways to be assigned to the DEGs, whereby 17 pathways were significantly enriched (*P* < 0.05, **Figure 4B** and **Supplementary Table S4**). The top 3 pathways in which the DEGs were primarily involved were steroid biosynthesis, extracellular matrix (ECM)–receptor interactions and the PPAR signaling pathway. The most significantly and uniquely enriched pathway for up-regulated DEGs was lipid acid metabolism and for down-regulated DEGs was steroid biosynthesis. Seven pathways related to fatty acid metabolism were also significantly enriched (**Supplementary Table S4**).

**FIGURE 5:**
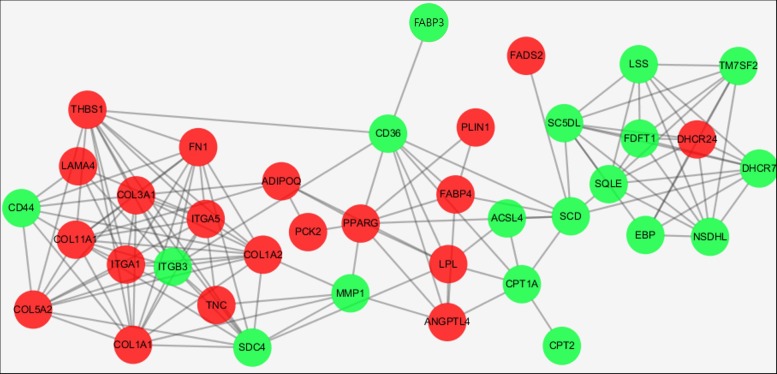
Protein interaction networks encoded by DEGs enriched in the top 3 KEGG pathways. Red and green points represent genes up-regulated and down-regulated, respectively.

We constructed a protein interaction network (**Figure 5**), in which integrin subunit beta 3 (ITGB3), collagen type I alpha 1 chain (COL1A1), collagen type XI alpha 1 chain (COL11A1), collagen type III alpha 1 chain (COL3A1), and collagen type I alpha 2 chain (COL1A2) were the most important interaction partners. Those proteins directly or indirectly interacted with several other DEGs and may be involved in regulatory cascades related to adipogenesis.

### Identification of TTR as Candidate Gene

**FIGURE 6:**
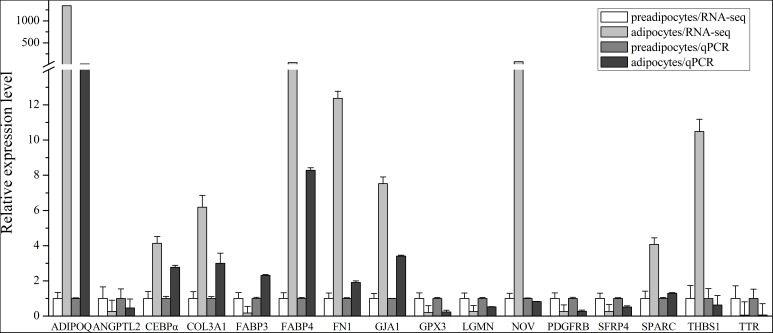
Relative expression level of 16 DEGs selected with high or low expression levels in RNA-seq data and qPCR detection. The expression level in preadipocytes is defined as “1.” In qPCR, all samples were measured in triplicate, and 4 preadipocyte samples and 4 adipocyte samples were used. The data represents mean ± SEM (Standard Error).

We screened the RNA-seq data for genes that were previously reported to be involved in preadipocytes differentiation, including LPL, FABP4, and CEBPα. As predicted, our RNA-seq results indicated that the expression levels of those genes increased during bovine preadipocyte differentiation. To validate the results obtained from RNA-Seq, we selected 16 DEGs and/or genes previously reported to be associated with adipogensis for qPCR, namely fibronectin1 (FN1) (Duarte et al., 2014), secreted protein, acidic and rich in cysteine (SPARC) (Rodríguez-Alvarez et al., 2010), collagen type III alpha 1 chain (COL3A1) (Duarte et al., 2014), angiopoietin like 2 (ANGPTL2), thrombospondin 1 (THBS1) (Blanco et al., 2012), TTR, legumain (LGMN), glutathione peroxidase 3 (GPX3), platelet derived growth factor receptor beta (PDGFRB), gap junction protein, alpha 1 (GJA1), nephroblastoma overexpressed (NOV), FABP3 (Boutinaud et al., 2013), adiponectin C1Q and collagen domain containing (ADIPOQ) (Zhang et al., 2014), secreted frizzled-related protein 4 (SFRP4) (Jeong et al., 2013), FABP4 and CEBPα, were selected for expression detection. All 16 genes showed significant expression changes between preadipocytes and adipocytes (*P* < 0.05) and 13 of them have the same expression trend as inferred from the RNA-seq analysis, while the expression trends of NOV, THBS1, and FABP3 in qPCR do not agree with those in RNA-seq data (**Figure 6**). In addition, the Pearson correlation between RNA-seq and qPCR data using these 13 genes for which the RNA-seq and qPCR measurements agree was calculated. It shows there is a significantly positively correlation between RNA-seq and qPCR data (*r* = 0.933, *P* < 0.01).

Among the DEGs validated by qPCR, the *ADIPOQ* gene and *TTR* gene showed the greatest changes of expression levels (*P* < 0.001, respectively), with almost 20-fold changes (**Figure 6B**). Since the effects of TTR during bovine preadipocyte differentiation has not been characterized yet, it was chosen as the candidate gene for subsequent experimentation.

### Involvement of TTR on Bovine Adipogenic Differentiation

**FIGURE 7:**
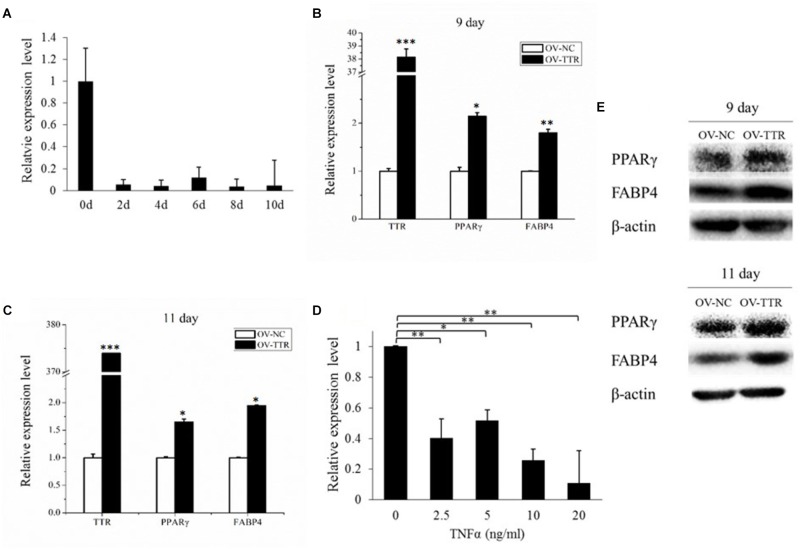
TTR is involved in bovine adipogenic differentiation. **(A)** The expression of TTR during bovine adipocyte differentiation. **(B)** On 9th day, overexpression of TTR increased mRNA expression of PPARγ and FABP4. **(C)** On 11th day, overexpression of TTR increased mRNA expression of PPARγ and FABP4. **(D)** TNFα treatment down regulate the expression of TTR. **(E)** TTR overexpression also promote protein expression of PPARγ and FABP4. Each treatment is in triplicate. OV-TTR: overexpression vector of bovine *TTR* gene. OV-NC: empty pcDNA3.1 (+) plasmid without any insertion fragment. ^∗^*P* < 0.05. ^∗∗^*P* < 0.01. ^∗∗∗^*P* < 0.001.

As soon as the preadipocyte was induced to differentiation, the expression level of TTR showed a significant decrease (**Figure 7A**). When we overexpressed TTR on 9 day after adipogenic induction, we observed strongly increasing mRNA (*P* < 0.05) and protein levels of PPARγ and FABP4 (**Figures 7B,E**). And on 11 day post-adipogenic induction, significant rising of expression levels of mRNA and protein of PPARγ and FABP4 were observed after overexpression of TTR (**Figures 7C,E**), suggesting that the overexpression of TTR promote bovine adipogenic differentiation. And we also found that overexpression of TTR slightly promotes the formation of lipid droplets (**Supplementary Figure S4**). Hence, TTR appears to be involved in the control of bovine aidpogenesis.

Tumor Necrosis Factor α (TNFα) is a cytokine that is associated with adipose tissue development (Arner et al., 2010). As shown in **Figure 7D**, increasing concentrations of TNFα significantly reduced the expression of TTR (*P* < 0.05), further corroborating an involvement of TTR in bovine adipogenesis.

## Discussion

Different types of adipose depots show distinct mechanisms of lipid accumulation (Fernyhough et al., 2005; Hausman et al., 2009). Most of animal’s storage lipids accumulate in the visceral and subcutaneous adipose tissue layer (Dodson et al., 2010). Subcutaneous fat depots determine meat quality, as the degree to which fat is stored in the subcutaneous fat layer correlates negatively with the extent of meat marbling (i.e., intramuscular fat deposition) and because subcutaneous fat itself serves as a quality assessment criterion (Jeremiah, 1996). Previous research related to the meat quality of beef cattle was motivated by the idea that reducing subcutaneous fat depots will bring about increased intramuscular fat depots (Underwood et al., 2008). Accordingly, previous research to establish a protocol for the isolation, culture, and induction of differentiation, of primary adipocytes typically used subcutaneous fat tissue samples, which are also comparatively easy to collect (Lengi and Corl, 2010; Song et al., 2010). Our present study reports on the culture of preadipocytes (and their differentiation into mature adipocytes) isolated from the subcutaneous fat tissue. In recent years, Ribo-Zero RNA-seq has been established as an efficient method to explore the transcriptional characteristics, e.g., during developmental processed (Adiconis et al., 2013; Zhao et al., 2014; Sun et al., 2015). Ribo-Zero RNA seq avoids rRNA interference and shows a high degree of technical reproducibility (Trapnell et al., 2012; Adiconis et al., 2013). In contrast to methods of RNA-Seq that prepare libraries based on poly-A enrichment, Ribo-Zero RNA-seq captures mRNA with or without a poly-A tails, allowing for more complete views on transcriptomic changes during development (Adiconis et al., 2013; Zhao et al., 2014; Sun et al., 2015). So our study provides large amounts of information for future studies on the regulatory mechanisms underlying adipogensis in beef cattle.

AS is an essential mechanism in post-transcriptional regulation and leads to protein diversity, which generates multiple different mRNAs and downstream proteins from a single gene through the inclusion or exclusion of specific exons (Pan et al., 2008; Burgess, 2012). Previous reports indicated that RI was the most common event in numerous species (Keren et al., 2010; Barbosa-Morais et al., 2012; Li et al., 2013; Wang et al., 2016). However, in this study, we found SE was the most common. It might be due to the reason that AS patterns varied across species, tissues types, and developmental stages (Keren et al., 2010; Barbosa-Morais et al., 2012; Li et al., 2013; Wang et al., 2016). Furthermore, a large number of genes found in this study underwent multiple types of AS differently during preadipocytes to adipocytes, indicating that AS may be involved in bovine adipocyte differentiation. The key transcription factors and growth factors functions in adipocyte differentiation, such as PPARγ, CEBP, IGF-1, and TGF-β, were not alternatively transcribed, which indicated that the effect of critical regulatory factors were highly conserved during bovine adipogenic differentiation, the previous study showed a similar result (Zhou et al., 2014; Zhang Y.Y. et al., 2017). Also, the expression levels of 817 AS isoforms were different among adipocytes and preadipocytes, which indicated a closely relationship between the cattle adipogenesis and AS. Therefore, AS may have an important role in bovine adipocyte differentiation.

Functional annotation of DEGs found a number of categories to be significantly enriched during bovine adipogenic differentiation. PPAR signaling pathway, ECM–receptor interaction, and Steroid biosynthesis were the top three KEGG pathways in which DEGs were involved. Those signal pathways were also significantly enriched in previous studies about RNA-seq of bovine adipose tissue (Zhou et al., 2014; Zhang Y.Y. et al., 2017). In our present study, we constructed a protein interaction network using DEGs and found that ITGB3, COL1A1, COL11A1, COL3A1, and COL1A2 were the most prominent interacted partners, highlighting the central roles played by collagen, a major component of ECM, during cattle adipogenesis. Recently, Ojima used protein sequencing during the differentiation of murine 3T3-L1 cell (Ojima et al., 2016). The author reported that ECM components were the most abundant secreted proteins secreted by differentiating adipocytes, along with a series of collagens, which matches the results of our present study (Trapnell et al., 2012). In our present study, however, expression level of these collagens were low during the early and middle differentiation, while a dramatic increase in the expression levels of most of the collagens was observed at 13th day of differentiation, representing a late stage of differentiation. Contrasting results could be attributed to species-specific differences of regulatory cascades or of adipose tissue formation and different time points at which samples were obtained. Finally, the protein interaction networks used in our present study were simplifications, prone to overlook a number of potential interaction partners. Collagens contribute to formation of fibril (Kofford et al., 1997; Gelse et al., 2003), however, future studies will be needed to provide deeper insights into the regulatory pathways related to bovine adipogenesis and the ECM by which the aforementioned proteins are linked to this process.

Among the 16 DEGs we selected for gene detection by qPCR, TTR showed the greatest change of expression, with a nearly 20-fold decrease during adipogenic differentiation. TTR is a carrier protein and it was hypothesized that TTR could transfer the active components from chylomicrons to adipocytes, thereby stimulating the acylation stimulating protein, a potent stimulator of adipocyte triacylglycerol storage in adipocytes (Scantlebury et al., 1998). Also, Matsuura found body fat mass is correlated with serum TTR levels in maintenance hemodialysis patients (Matsuura et al., 2017). In our study, we found overexpression of TTR to promote PPARγ and FABP4 expression during bovine adipocyte differentiation, and to promote the formation of lipid droplets. Previous study showed that TTR was shown to increase 10-fold after 24 h overexpression of FABP4 and decrease to nearly zero after 48 h overexpression of FABP4 during the adipogenic differentiation of bovine skeletal muscle stem cell (Zhang L. et al., 2017), which also shows there were a relationship between the expression of TTR and FABP4 during adipogenic differentiation. On the other hand, we found a significant decreasing expression of TTR after mature adipocytes were treated with TNFα, a factor known to contribute to the development of adipose tissue. Altogether, our results suggest that TTR may function as a stimulator of gene that drives bovine adipocyte differentiation. In RNA-seq data, we found no significant increase of PPARγ and CEBPα after adipogenic induction. PPARγ and CEBPα are two major transcription factors regulating adipogenic differentiation. Their expression dramatically increased after adipogenic induction (Chawla et al., 1994; Darlington et al., 1998). At a late differentiation stage, such as 13th day, they have already reached the peak expression level and then decrease to a level without significant difference (Takenaka et al., 2013; Hu et al., 2015). Even though, their expression levels in adipocytes are still higher than that in preadipocytes. On the other hand, as methods used to detect mRNA expression, qPCR and RNA-seq are quite different, similar with our study, other reports showed different expression patterns of the same genes between RNA-seq and qPCR (Marioni et al., 2008; Wagner et al., 2012).

## Conclusion

In conclusion, using Ribo-Zero RNA-seq, our study is to provide an overview of transcriptome changes during adipogenesis, namely, during the differentiation of preadipocytes into mature adipocytes. Hundreds of DEGs related to bovine adipogenesis were detected, only few of which could be further characterized regarding their mechanistic involvement during adipogenesis. AS may have an important effect during bovine adipocyte differentiation. Based on the top three enriched KEGG pathways, collagens that are associated with ECM might play central roles in cattle adipocyte differentiation. More importantly, the potential regulatory effect of TTR during bovine adipocyte differentiation is proposed. Our study leaves an array of new questions related to the molecular mechanisms underlying the regulation of bovine adipocyte differentiation and provides primary information for further functional studies about TTR in bovine adipogenesis.

## Data Availability

The raw transcriptome read data generated from this study has been deposited into NCBI Short Read Archive (SRA) under accession number SRP067820.

## Author Contributions

HFC designed and performed the experiments, and wrote the paper. ML designed the experiments and wrote the paper. XS analyzed the data and helped to design the experiments. MP helped the paper writing and language correction. CJL corrected language and the paper design. XL discussed the experiment design. CZL helped to design the experiment. YH helped to analyzed the data. YB provided the samples. XQ collect the samples. FL discussed the experiment design. HC helped to the experiment design and the paper writing.

## Conflict of Interest Statement

The authors declare that the research was conducted in the absence of any commercial or financial relationships that could be construed as a potential conflict of interest.

## Footnotes

1 http://ftp.ncbi.nlm.nih.gov/genomes/Bos_taurus/Assembled_chromosomes/seq

2 http://www.geneontology.org/

3 http://www.kegg.jp
