# Supplementary material for: Global Transcriptome Analysis During Adipogenic Differentiation and Involvement of Transthyretin Gene in Adipogenesis in Cattle
Source: Front Genet. 2018 Oct 18;9:463. doi: 10.3389/fgene.2018.00463 (PMC6200853; doi:10.3389/fgene.2018.00463)
Supplement: Supplementary file 2 [file Table_2.DOCX]

**Global transcriptome analysis during adipogenic differentiation: effects of the transthyretin gene on adipocyte differentiation in cattle**

**Hanfang Cai, Mingxun Li, Xiaomei Sun, Martin Plath, Congjun Li, Xianyong Lan, Chuzhao Lei, Yongzhen Huang, Yueyu Bai, Xinglei Qi, Fengpeng Lin, and Hong Chen**

**Correspondence author: HONG CHEN, E-mail: chenhong1212@263.net**

**
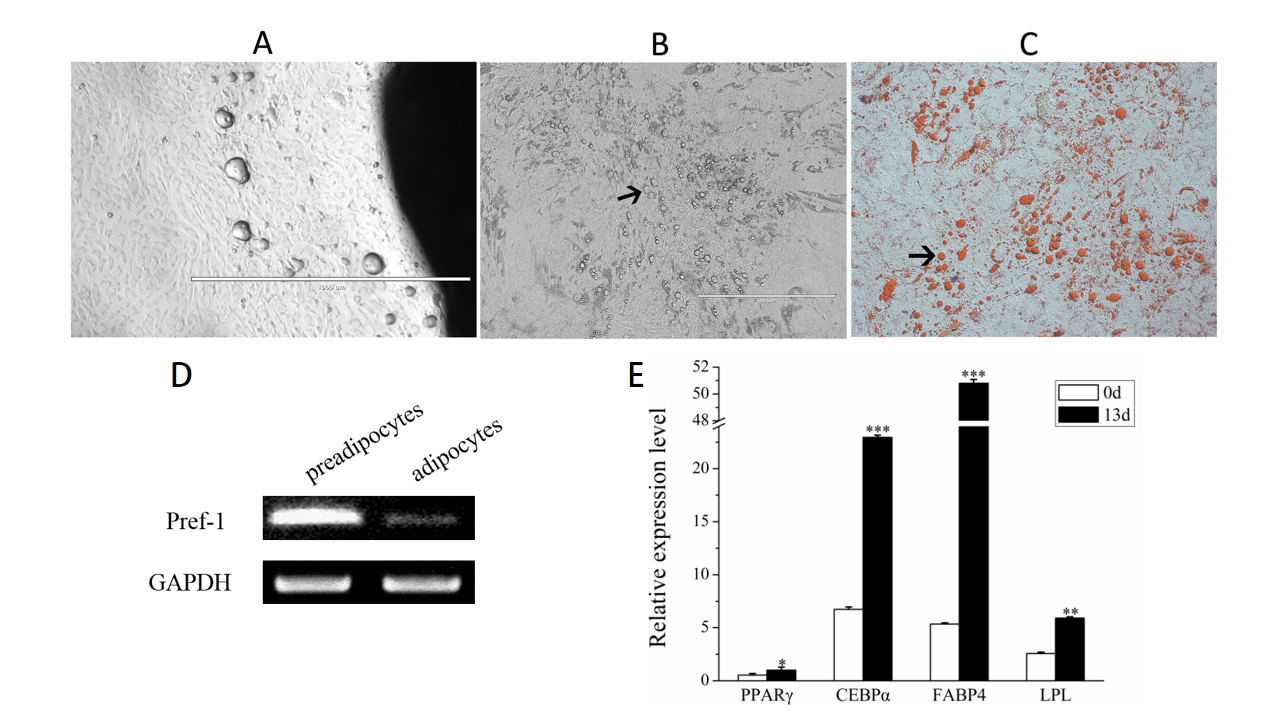
**

**Figure S1.** Adipocyte identification. (A) Primary adipocytes moved from bovine adipose tissue, and reached at the confluence of 100% at 8 d. The black part was adipose tissue. Scale bar: 1000 μm. (B) Morphology of bovine differentiated adipocytes on day 13. Scale bar: 400 μm. (C) Bovine differentiated adipocytes stained by Oil Red O on day 13. The magnification was × 200. The black arrow indicated one of the lipid droplets. (D) Reverse transcriptional PCR (RT-PCR) analysis of Pref-1 expression in preadipocytes and adipocytes. *GAPDH* gene was used as control. (E) The expression of adipogenic specific genes, including PPARγ, CEBPα, FABP4 and LPL, were detected in preadipocytes (0d) and differentiated adipocytes (13d). * *P*<0.05, ** *P*<0.01, ****P*<0.001.

**
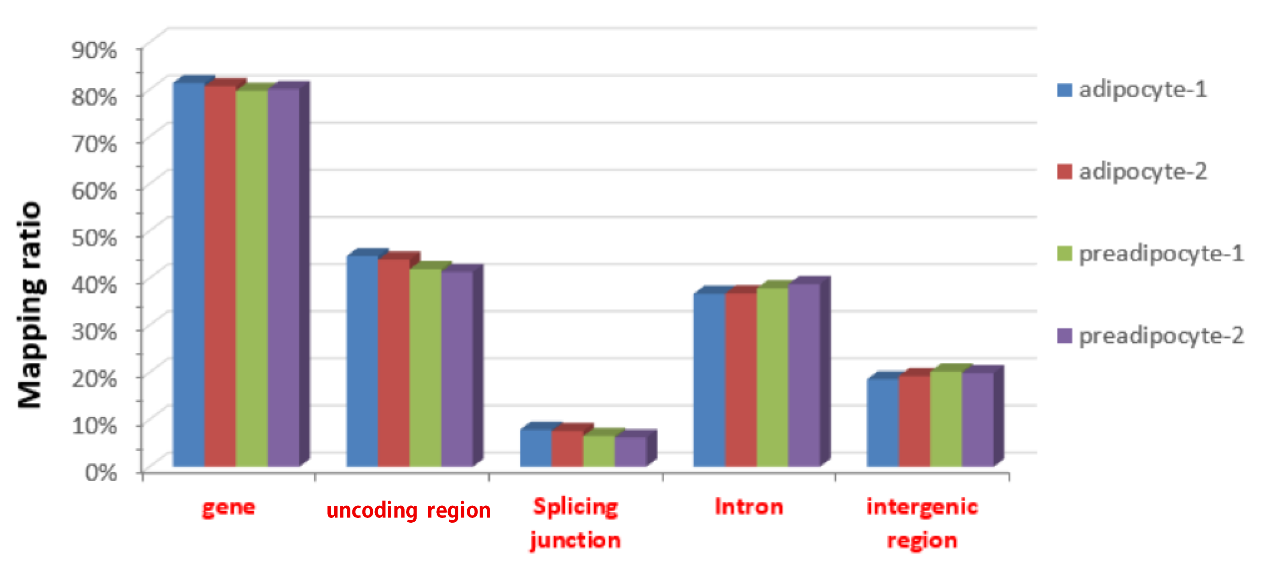
**

**Figure S2.** Reads mapping region distribution. Non-coding region concludes 5’ untranslated region, 3’ untranslated region and non-coding RNA regions.


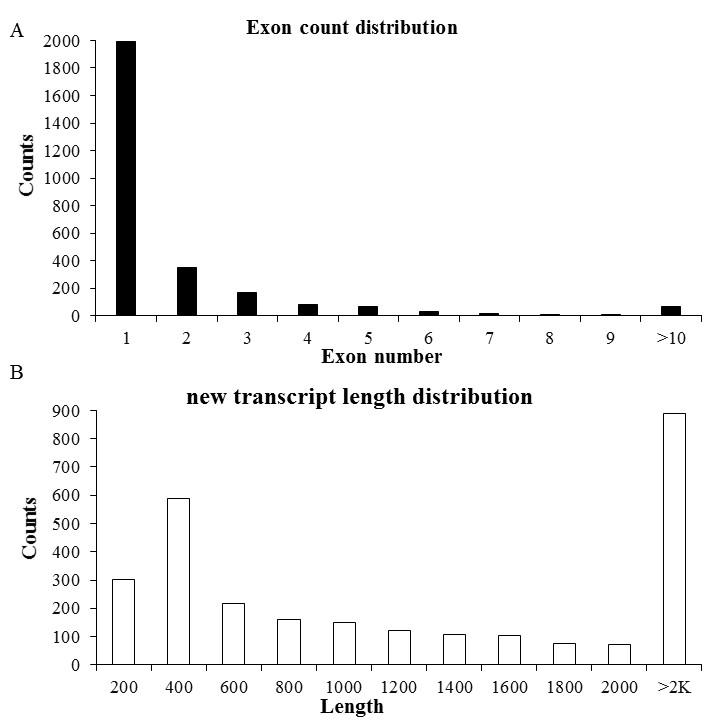


**Figure S3.** The exon count distribution (A) and length distribution (B) of new genes.


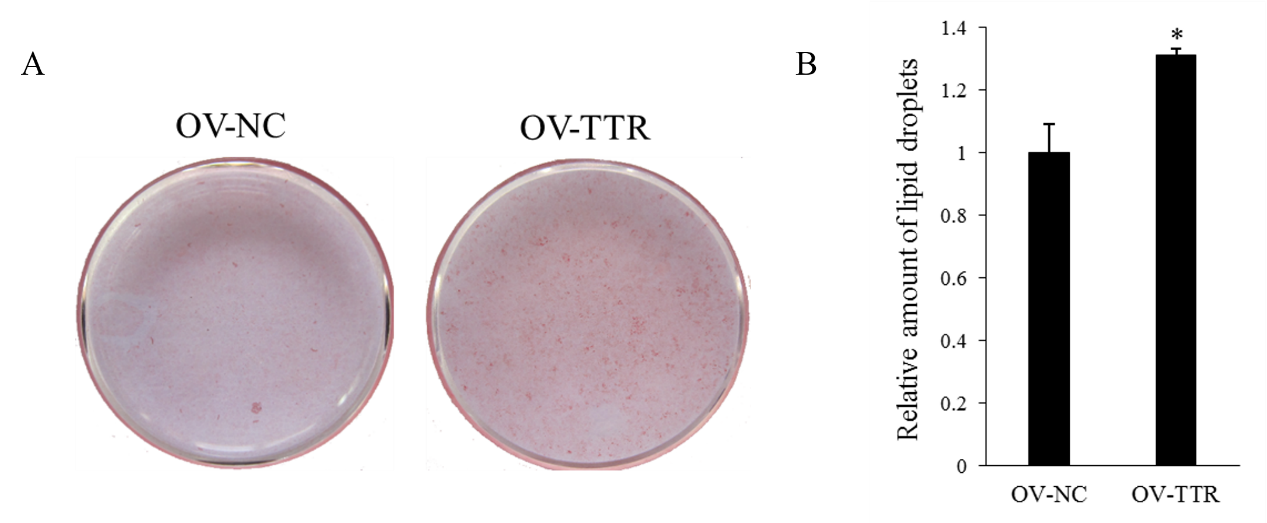


**Figure S4**. Overexpression of TTR promote the formation of lipid droplets. (A) Oil Red O staining was used to evaluate the formation of lipid droplets. (B) Relative amount of lipid droplets were measured by UV spectrophotometer at 490 nm. The value of OV-NC was defined as “1”. * *P* < 0.05

**Table S5 Primers for qPCR of 16 DEGs selected with high or low expression level**

| Gene | Primer sequence (5’-3’) | Product length (bp) |
| --- | --- | --- |
| *Pref-1* | F: ATGGCTTCCTTCCGTCTGTTC  R: CACCAAATCCTGTCCTTCATCC | 491 |
| *FN1* | F: GCGTGTCACCTGGGCTCCAC  R: CGGTGCCGGGCAGGAGATTT | 149 |
| *SPARC* | F: GAATTCGATGATGGTGCTGAGG  R: GTATTTGCAAGGCCCGATGTAG | 294 |
| *COL3A1* | F: GGCCCCCTGGAAAGGACGGA  R: CCCCGCCAGCACCACAACAT | 168 |
| *ANGPTL2* | F: CCACGATGTCTACACAGGCAA  R: CACCACCTTCTTGAGCGAGT | 181 |
| *THBS1* | F: GGCACAAATAGCTCCACCAT  R: AGCTCCGGTGAGTTCAAAGA | 135 |
| *TTR* | F: CCTCGGGGAAAACCAGTGAA  R: TTCATGGAACGGGGAGATGC | 134 |
| *LGMN* | F: GCTCACTTCTTTGAGGCTGC  R: CATCTGCCTGGTGCCTGTAA | 227 |
| *GPX3* | F: TTGGTCTGGTCATTCTGGGC  R: CGAACATACTTGAGGGTGGCT | 94 |
| *PDGFRB* | F: AGGCATCAGCAGCAAGGATA  R: CAGGTCAGGACAAAGGTGCT | 183 |
| *GJA1* | F: GTGCCTGGGCTTGCTTTTTC  R: GCCTGGGTACTGCTCTTTCT | 100 |
| *NOV* | F: CCAGACCCTTCCAGCCTACA  R: TGCCACAGCTCTTGGAACAT | 116 |
| *FABP3* | F: GCGTTCTCTGTCGTCTTTCCC  R: CTGTGTTCTTGAAGGTGCTTTGTG | 224 |
| *Adiponectin* | F: GGCATTCCAGGGCATCCT  R: TTCCAGTTTCACCAGTGTCAC | 130 |
| *SFRP4* | F: AGGAGTGGCTGTAATGAAGTAAC  R: TTGATGAGGCAGGATGTGTG | 135 |
| *FABP4* | F: AAGTCAAGAGCATCGTAA  R: CCAGCACCATCTTATCAT | 111 |
| *CEBPα* | F: CAAGAACAGCAACGAGTA  R: GTCATTGTCACTGGTCAG | 130 |
| *Gapdh* | F: GGTCACCAGGGCTGCTTTTA  R: CCAGCATCACCCCACTTGAT | 222 |
